# Delivering co-stimulatory tumor necrosis factor receptor agonism for cancer immunotherapy: past, current and future perspectives

**DOI:** 10.3389/fimmu.2023.1147467

**Published:** 2023-04-25

**Authors:** Osman Dadas, Ayse Ertay, Mark S. Cragg

**Affiliations:** ^1^ Antibody and Vaccine Group, School of Cancer Sciences, Faculty of Medicine, University of Southampton, Southampton, United Kingdom; ^2^ School of Cancer Sciences, Faculty of Medicine, University of Southampton, Southampton, United Kingdom; ^3^ Institute for Life Sciences, University of Southampton, Southampton, United Kingdom

**Keywords:** TNFR, agonism, co-stimulation, cancer, immunotherapy

## Abstract

The tumor necrosis factor superfamily (TNFSF) and their receptors (TNFRSF) are important regulators of the immune system, mediating proliferation, survival, differentiation, and function of immune cells. As a result, their targeting for immunotherapy is attractive, although to date, under-exploited. In this review we discuss the importance of co-stimulatory members of the TNFRSF in optimal immune response generation, the rationale behind targeting these receptors for immunotherapy, the success of targeting them in pre-clinical studies and the challenges in translating this success into the clinic. The efficacy and limitations of the currently available agents are discussed alongside the development of next generation immunostimulatory agents designed to overcome current issues, and capitalize on this receptor class to deliver potent, durable and safe drugs for patients.

## Introduction

Members of the tumor necrosis factor superfamily (TNFSF) and their receptors (TNFRSF) are important regulators of the immune system. Interaction between these ligands and receptors can mediate proliferation, survival, differentiation, and function of immune cells (1, 2). There are 19 TNFSF ligands and 29 TNFRSF receptors, representing a large and diverse family.

**Figure 1:**
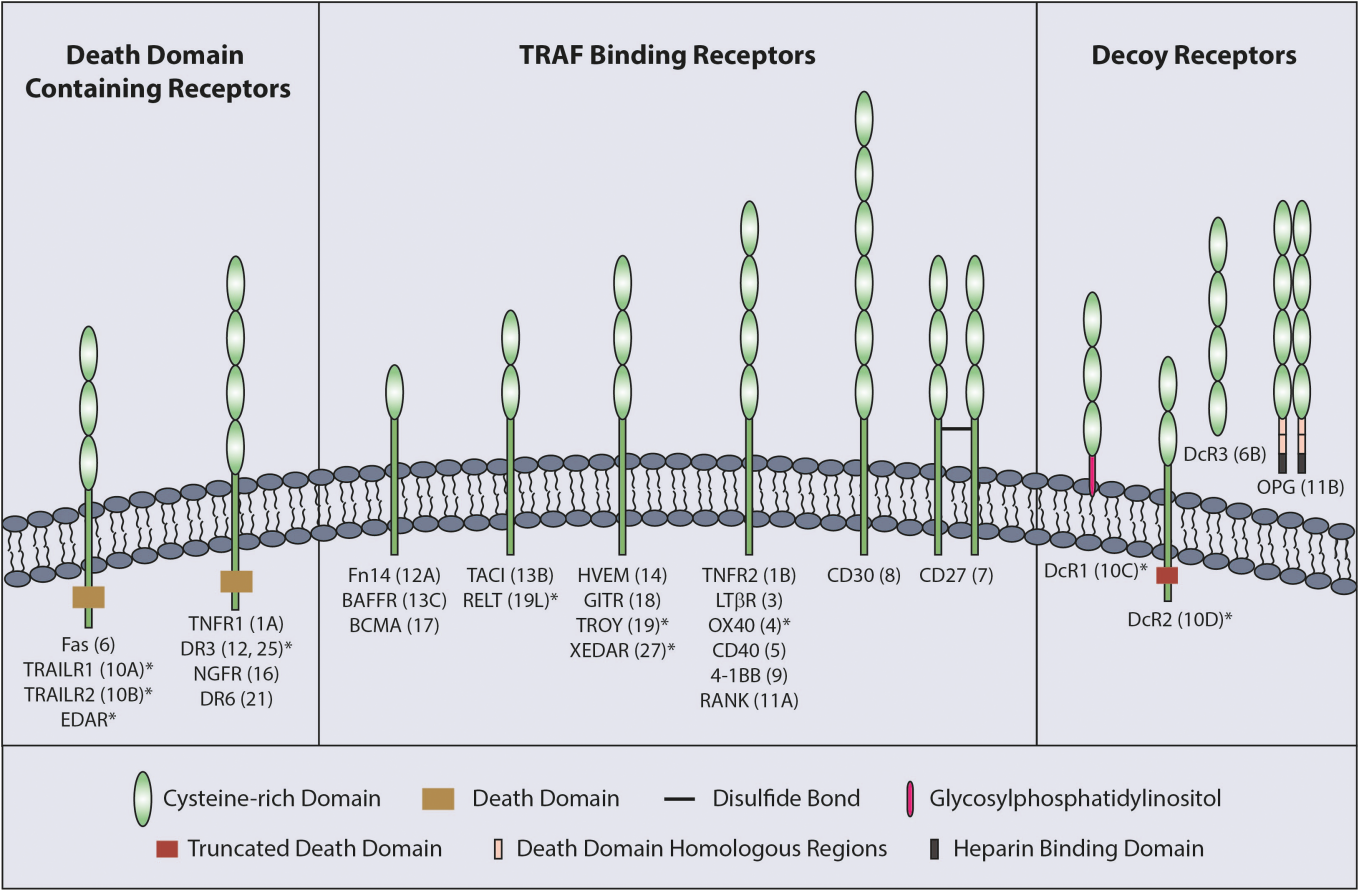
Classification of TNFRSF. The TNFRSF can be classified into three sub-families. All twenty-nine members of the family, grouped into three sub-families, are indicated with the number of CRDs on their extracellular region and TNFRSF number in brackets. CRD domains are defined by Uniprot with the exception of RELT which was published as having two CRDs (4). * indicates the receptors with a truncated CRD domain.

The TNFSF ligands are type II proteins which are characterized by the presence of a C-terminal TNF homology domain (THD) responsible for ligand trimerization and receptor binding (3). In comparison, the TNFRSF receptors have between one to six cysteine rich domains (CRD) in their extracellular region (**Figure 1**) that are involved in ligand binding and receptor auto-association (5).

TNFRs can be sub-divided into three groups according to functional and structural differences; death domain (DD) containing receptors, decoy receptors and TNF receptor associated factor (TRAF) binding receptors (**Figure 1**). The DD is an 80 amino acid domain present in the cytoplasmic tail of the DD containing receptors. Although the DD containing receptors mainly initiate cell death signaling, they can also mediate other outcomes, such as NF-κB signaling (1, 5). The decoy receptors lack signal initiation capacity and consist of glycosylphosphatidylinositol (GPI) tethered receptors, soluble receptors and receptors possessing a non-functioning DD (5). Finally, TRAF binding receptors possess TRAF-interacting motifs (TIF) in their cytoplasmic tail that is responsible for recruiting TRAFs to mediate downstream signaling upon receptor activation.

Following expression on the cell surface, several members of the TNFRSF can self-associate into dimers or multimers prior to ligand binding. Although some members can be found as covalently linked dimers (e.g. CD27 (6)), self-association for others is mainly driven by the pre-ligand assembly domain (PLAD), largely covering the N-terminal CRD1 (7, 8), and glucocorticoid-induced TNFR related protein (GITR) is an exception as dimerization of this TNFR is driven by interactions within CRD3 (9). Formation of receptor dimers or trimers for several members of the TNFRSF before ligand binding has been shown to be crucial for their interaction with ligand. Deletion of the PLAD domain in TNFR1 and TNFR2, significantly reduced TNFα binding to both receptors (7, 10). Although the ligand binding domain is located in CRD2/3, the reduced binding suggested that ligand-independent multimerization, driven by the PLAD domain, is important for ligand binding.

The TNFSF ligands can be found in soluble or membrane bound forms. Although one group of TNFRSF members (category I) can be activated by soluble ligand trimers, others (category II) require interaction with the membrane bound ligand to be fully activated (5). For example, soluble TNFα binds with higher affinity to TNFR1 than TNFR2 and primarily activates TNFR1 signaling, whereas TNFR2 is mainly activated by membrane bound ligand (11, 12). Although CD40 and GITR are both activated by trimeric ligands, activation is further enhanced with higher valency ligands or cross-linking of the trimeric molecules, presumably through induction of higher-order clustering (9, 13, 14). In contrast, CD27 and 4-1BB show minimal activation and require higher-order clustering (13). As described above activation of TNFRSF members can lead to multiple cellular outputs including proliferation, survival and differentiation, several of which may be therapeutically beneficial.

## Rationale behind targeting TNFRSF

In addition to T-cell receptor (TCR) interaction with peptide-MHC (major histocompatibility complex), T cells require co-stimulatory signaling to be fully activated and generate an optimal response (15). Co-stimulatory TNFRSF members expressed on T cells include CD27, OX40, 4-1BB, TNFR2 and GITR. Co-stimulatory receptors on antigen presenting cells (APCs) are also important, with molecules such as CD40 playing a critical role in licensing and activation of dendritic cells (DCs) and B lymphocytes during an immune response (16), to elicit appropriate humoral and cellular adaptive immunity. DCs can be excluded from the tumor microenvironment and multiple immunosuppressive mechanisms can suppress their maturation and full activation, preventing effective T-cell responses (17–19). The DCs up-regulate multiple TNFSF ligands after maturation which are required for the optimal co-stimulation of T cells. Thus, targeting the TNFRSF members to provide co-stimulation is an attractive approach to elicit effective T-cell responses.

The majority of the T-cell co-stimulatory receptors are only upregulated and appreciably expressed after TCR activation, e.g. 4-1BB expression on adoptively transferred T cells is detected 12 to 24 hrs after stimulation (20) whereas others, most notably CD27, are constitutively expressed on T cells (2). Once expressed, the various TNFR are available for engagement by their ligands, which themselves also possess specific kinetics of expression (21). Although the downstream signaling pathways of the co-stimulatory TNFRSF members are not identical, signals are mainly initiated after TRAF recruitment to their cytoplasmic tails which leads to NF-κB and JNK pathway activation (22).

Stimulation of these co-stimulatory receptors contributes to enhanced effector function but also survival of the T cells. For instance, CD27 stimulation through engagement of its ligand CD70 leads to expression of cytokines such as IFN-γ, Interleukin-12 (IL-12), IL-5, IL-4 and IL-2 (2, 23), alongside the complementary cytokine receptors including IL-12R and IL-2R. Similar to CD27, stimulation of OX40 leads to upregulation of cytokines and cytokine receptors such as IL-12R and IL-2R on T cells, supporting their activation (24, 25). GITR stimulation also promotes the expression of IFN-γ, IL-2 and IL-2R (26) and is required for optimal CD8^+^ effector T-cell generation as absence of GITR on CD8^+^ T cells significantly reduces their expansion following an influenza infection (27). CD27 engagement can alter cellular metabolism to support the rapid expansion of T cells after activation. Here, the expression of the serine threonine kinase Pim-1 is upregulated to facilitate increased aerobic glycolysis and protein translation during proliferation (28–30).

TNFR signaling also supports survival of activated T cells. CD27 increases expression of the anti-apoptotic protein Bcl-XL in T cells, reduces the level of FasL on CD4^+^ T cells and reduces CD8^+^ T-cell sensitivity to FasL-stimulated apoptosis (29, 31). Similarly, anti-apoptotic proteins such as Bcl-XL and Bcl-2 are upregulated following OX40 stimulation (32), Bcl-XL and Bfl-1 are upregulated by 4-1BB (33) and Bcl-XL is upregulated after GITR engagement (27).

CD27 signaling induces CD8^+^ T-cell differentiation into cytotoxic T lymphocytes (CTL) and CD4^+^ T-cell differentiation into Th1 cells (28). Increased cytotoxic capacity of CTLs is supported by mechanisms such as upregulated of IL-2, important for their survival, and IFN-γ, which is further upregulated by IL-2 signaling. Increased cytotoxic capacity and effector functions of CD8^+^ T cells has also been shown after 4-1BB stimulation (34). Similar activities are evident on APCs, where CD40 signaling is critical for their ability to induce effective CD8^+^ T-cell responses. Stimulation of CD40 on DCs is important for their maturation and ability to present antigens to T cells. Activation of CD40 also leads to production of pro-inflammatory cytokines such as IL-12, IL-6 and IL-1β (35). Moreover, CD40 stimulates expression of co-stimulatory ligands such as CD80 and CD86, that interact with the receptors on T cells (e.g. CD28) for further activation.

In addition to the effects during naïve T-cell priming, co-stimulatory receptors of the TNFRSF contribute to the generation of the memory T-cell pool. CD27 signaling during the initial activation phase of CD8^+^ T cells is required for the development of memory CD8^+^ T-cell subsets and efficient expansion during the secondary response. Stimulation of CD27 during the initial response leads to IL-7Rα expression on effector CD8^+^ T cells, which in turn increases the frequency of memory precursor cells (36, 37). Similarly, 4-1BB and OX40 signaling are required for the generation of robust memory T-cell pools (38, 39). Stimulation of antigen specific CD8^+^ T cells with a 4-1BB agonist during priming leads to the generation of a strong memory CD8^+^ T-cell pool, resulting in a high secondary response (40). OX40 signaling is also important for T-cell memory. Although the primary expansion of CD8^+^ T cells was not impaired in OX40L-/- mice following influenza infection, there were defects in the secondary response of the virus specific CD8^+^ T cells (41). GITR has also been shown to be important for the secondary expansion of memory CD8^+^ T cells as *in vitro* generated WT or GITR-/- memory cells showed significantly different expansion capacity in an influenza infection recall response (27).

Additionally, the crucial role of co-stimulatory members of the TNFRSF in generating immune surveillance is evidenced by the development of various pathologies in individuals with TNFR deficiencies/mutations. For example, deficiency of CD27 or CD70 can lead to development of Epstein-Barr virus (EBV)-related immunodeficiency and lymphoproliferative disorders including B-cell malignancies (42, 43). Characterization of the immune response of an individual with CD27 deficiency who had hypogammaglobulinemia and persistent symptomatic EBV viremia revealed impaired IL-2 production in their CD8^+^ T cells which are the primary immune cells responsible for clearing EBV infections. IL-2 is critical for CD8^+^ T-cell function and impaired IL-2 production contributes to defective immune responses (42, 44, 45). 4-1BB deficiency can also lead to EBV driven complications and individuals can have persistent EBV viremia and EBV-related lymphoproliferation. CD8^+^ T cells from 4-1BB deficient individuals showed reduced proliferative and cytotoxic capacity (46). Deficiency in functional OX40 can lead to Kaposi sarcoma development in individuals with human herpes virus 8 infection (47). Similarly, CD40 or CD40 ligand deficiency can lead to immunodeficiency due to impaired APC function, which subsequently leads to impaired T-cell responses (48, 49), alongside an absence of germinal center-mediated somatic hypermutation and class switching in the humoral response known as hyper-IgM syndrome (50, 51). Dysregulation of the TNFRSF co-stimulatory receptor signaling and associated diseases identified to date are illustrated in **Table 1**. Further, the importance of co-stimulatory TNFRSF members in functional immune response generation is also supported in multiple constitutive and conditional TNFRSF knock out (-/-) models. For example, 4-1BBL deficiency in mice leads to impaired CD8^+^ T-cell responses against viral infections and predisposes the mice to B-cell lymphoma development (72–74). Similarly, CD27-/- mice have defects in the generation and accumulation of effector T cells at the site of infection following influenza infection, with the memory T-cell pool impaired (75, 76).

**Table 1:** TNFRSF co-stimulatory receptor dysregulation and disease development.

Receptor	Defect	Associated disease	Reference
**CD27**	Absent or reduced receptor expression	EBV related immunodeficiency B-cell malignancies	(42, 52–54)
**4-1BB**	Absent receptor expression	EBV-related lymphoproliferation	(46)
**OX40**	Reduced receptor expression and defective ligand binding	Kaposi sarcoma after human herpes virus 8 infection	(47)
**CD40**	Defective receptor expression or defective ligand binding	Impaired T-cell responses Hyper-IgM syndrome	(48, 55, 56)
**TNFR2**	Gene polymorphisms (Effects on the receptor not yet characterized)	Autoimmune diseases Hepatitis B virus related liver disease	(57, 58) (59)
**BAFFR**	Loss of function mutation Gain of function mutation	Common variable immunodeficiency Non-Hodgkin lymphoma Autoimmunity	(60, 61) (62) (63)
**TACI**	Defective receptor expression, defective ligand binding or defective signaling	Common variable immunodeficiency IgA deficiency	(64–66)
**HVEM**	Absent or reduced receptor expression	B-cell malignancies	(67–69)
**RELT**	Loss of function mutations or mutations predicted to reduce protein stability	Amelogenesis Imperfecta	(70, 71)

These co-stimulatory receptors have been reported to contribute to a clinical condition as a consequence of defects in their normal expression, function or ligand binding.

As the importance of co-stimulatory TNFRSF members in the development of a functional immune response has become clear, many of these receptors have subsequently been targeted to modulate the immune response in the context of immunotherapy. In this review we have restricted ourselves to discussing findings mainly in the field of cancer immunotherapy. Moreover, as various definitions of agonism exist, here we have defined agonism as activating the target receptor either *via* Fc gamma receptor (FcγR) dependent or independent mechanisms.

## Therapeutic targeting of the TNFRSF

Agonistic targeting of the co-stimulatory members of the TNFRSF has shown to be effective in pre-clinical tumor models. Targeting 4-1BB in tumor models representing liver cancer, floor of mouth squamous cell cancer, colorectal cancer and lymphoma, using monoclonal antibodies (mAb) or recombinant 4-1BBL has generated robust anti-tumor responses (77–80). Buchan et al. demonstrated that two different mechanisms can contribute to a robust anti-tumor response induced by anti-4-1BB antibodies in certain models and contexts; 1) stimulating the effector T cells and 2) depleting T regulatory (Treg) cells. Additionally, depleting Tregs first and then agonizing the effector T cells induced better responses than only depleting the Tregs or agonizing the effector T cells (79). Similar to 4-1BB, targeting OX40 or GITR has been shown to stimulate robust anti-tumor responses in several pre-clinical tumor models, through a similar mechanism of action i.e. agonizing effector T cells or depleting Tregs (81–83). Treatment of solid tumors in a pre-clinical study with an agonistic anti-GITR mAb, increased the infiltration and activity of effector CD4^+^ and CD8^+^ T cells (84). In another study with the same agonistic mAb targeting a different solid tumor model however, the effect was mainly through depletion of intra-tumoral Tregs and slight increase in the infiltration of CD8s which resulted in a significantly improved CD8^+^ to Treg ratio (85). Additionally, the CD8^+^ T cells exhibited a more activated phenotype. These results indicate that anti-GITR mAbs can also act through different mechanisms and the dominant mechanism of action can vary depending on the tumor model. Moreover, it has been shown for OX40 and GITR targeting that the differential level of expression on effector T cells vs Tregs can lead to preferential depletion of Tregs as a consequence of higher levels of receptor expressed on them, enhancing immunotherapy (86–88).

Targeting CD27 has also been shown to induce significant anti-tumor responses in several pre-clinical models. Agonistic anti-CD27 antibody was efficacious in murine lymphoma models such as BCL_1_ and A31 (89). In a study where DCs in CD27-/- mice were manipulated to exhibit constitutive expression of CD70, an ovalbumin (OVA) expressing melanoma model (B16-OVA) was rejected following OVA specific (OT-1) CD8^+^ T cell transfer and OVA challenge whereas adoptive transfer of CD27-/- OT-1 CD8^+^ T cells did not elicit protective anti-tumor immunity (90) indicating the contribution of CD27/CD70 pathway to anti-tumor response in this model. In theory, targeting CD27 can induce anti-tumor responses by either agonizing the effector cells or depleting the Tregs dependent on the level of expression on individual cell populations (91) similar to targeting other members of the TNFRSF. Additionally, the method of CD27 targeting (modality, engagement of FcγR etc.) is also a key issue determining the mode of action as described in more detail below. Despite providing a strong anti-tumor response, the most agonistic anti-CD27 mAb also induced activation induced cell death in the effector CD8^+^ T cells (91) indicating that the strength of the stimulation needs to be appropriately tuned to induce a strong primary immune response and not impair other effects such as memory generation.

Another therapeutically exciting TNFR, TNFR2, is expressed on multiple immune cells, including Tregs at high levels and has been shown to be crucial for their survival. Therefore, targeting TNFR2 to deplete Tregs was considered as a potential mechanism to boost effector T-cell responses in anti-tumor immunity. Although several studies demonstrated the possibility of such an approach (92, 93), it has recently been shown that targeting TNFR2 can also work through agonistic mechanisms in pre-clinical models. Tam and colleagues demonstrated that an agonistic anti-TNFR2 mAb could stimulate the expansion of tumor specific CD8^+^ T cells with improved effector function. The agonistic mAb was efficacious in multiple pre-clinical solid tumor models and agonizing the effector CD8^+^ T cells was shown to be the main mechanism of action as demonstrated by increased frequency and functionality of antigen specific CD8^+^ T cells without the depletion of Tregs (94).

## Antibody targeting of the TNFRSF

**Figure 2:**
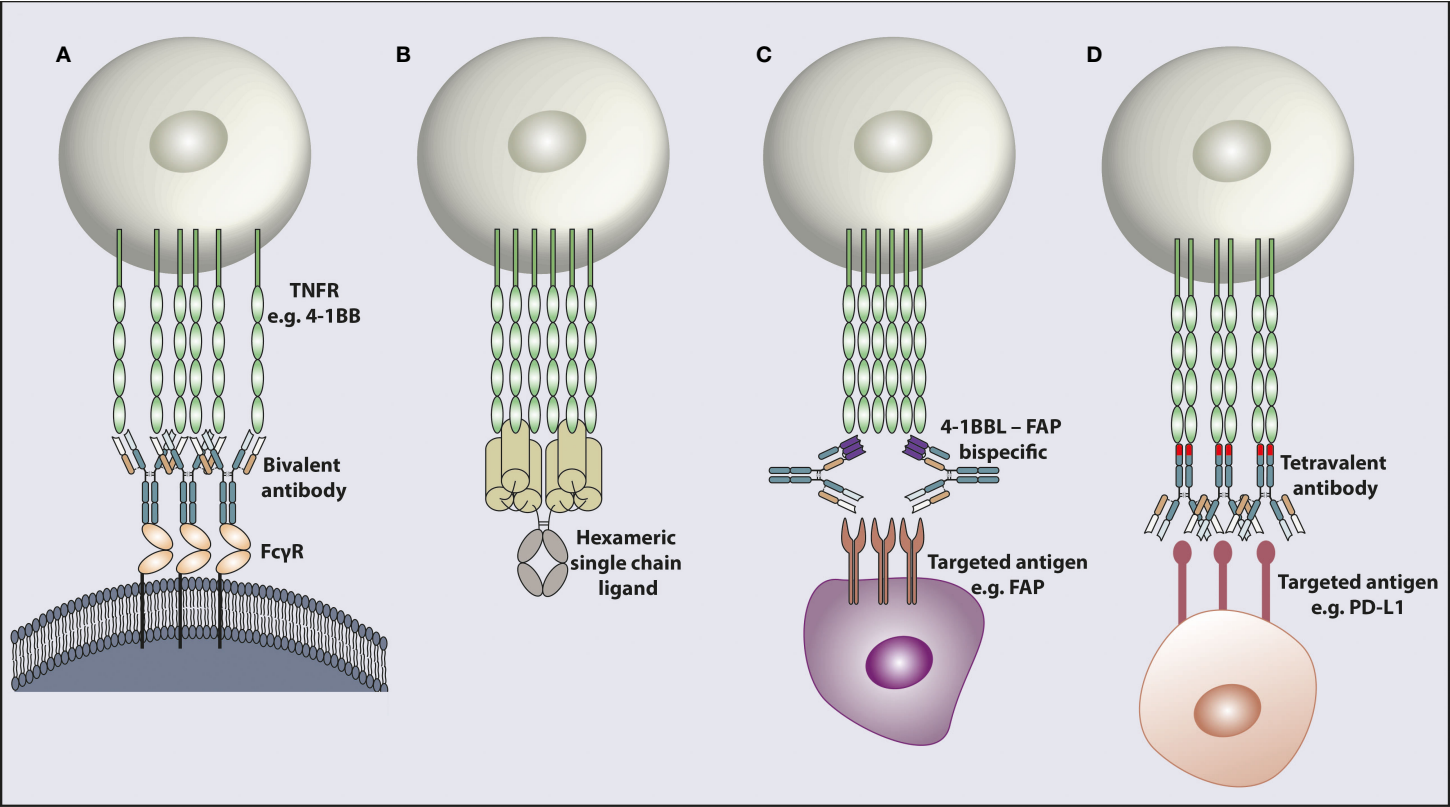
Modalities for targeting and activating TNFR. TNFR cross-linking achieved by different mechanisms. **(A)** Engagement with FcγR enables bivalent mAb cross-linking leading to target receptor clustering. **(B)** A recombinant hexameric single chain ligand inducing receptor clustering. The hexameric ligand structure is composed of a full Fc domain and six TNFSF ligand ECDs. **(C)** An antibody shaped bispecific molecule with one antigen binding arm targeting a TNFR e.g. 4-1BB and the other arm targeting a receptor e.g. FAP in the tumor microenvironment. **(D)** A bispecific molecule in a tetravalent format with two antigen binding arms targeting one receptor e.g. PD-L1 and the other two antigen binding arms in the opposite end of the molecule binding the TNFR e.g. 4-1BB, to induce receptor clustering. The 4-1BB binding domains inserted into the CH3 domain are indicated as a different color in the CH3 domain. TNFR; tumor necrosis factor receptor, FcγR; Fc gamma receptor, 4-1BBL; 4-1BB ligand, FAP; fibroblast activation protein, PD-L1; programmed death ligand 1.

The main method for targeting the TNFRSF to date has been by using mAb. As the TNFRSF members require trimerization and higher-order clustering for optimal activation, one way that canonical bivalent mAbs can achieve this is by concurrently engaging with FcγR (**Figure 2A**). Depending on their isotype and subclass, mAbs interact with different FcγRs (95, 96). In mouse models, the mIgG1 isotype interacts with the inhibitory FcγRIIB with higher affinity and mediates further TNFR clustering to induce strong agonistic responses. However, depending on the tumor model, anatomical location of the tumor and microenvironmental factors, the availability of FcγRIIB can be limiting, impacting the response. In support of this observation, it has been shown in pre-clinical studies that the agonistic activity of anti-CD40 and anti-4-1BB mIgG1 antibodies relies on the availability of FcγRIIB (79, 97). It was further demonstrated that a two-fold reduction in FcγRIIB expression completely eliminated the agonistic activity of certain agonist anti-TNFR mAbs *in vivo* (98). *In vitro* studies support that for CD40 at least, if expressed at sufficient level, all FcγR can mediate increased agonism in line with their relative affinities for the given mAb isotype (97, 99). Cross-linking of the receptors is the most likely explanation for mAb induced agonism with chemical cross-linking of a mIgG2a mAb able to elicit potent agonism *in vivo*, whereas the native mIgG2a does not (100). Importantly, several studies have shown that downstream signaling from FcγRIIB is not required for its cross-linking activity (97, 98), most recently demonstrated for OX40 mAb in a mouse expressing FcγRIIB with a mutant, non-signaling, ITIM (101). Therefore, why FcγRIIB has this key cross-linking role in mice is not fully clear but perhaps relates to expression in the right place at the right time and the fact that multiple mouse models upregulate FcγRIIB in the tumor microenvironment, potentially due to hypoxia (102). Other variables such as the genetic background of the mouse strain may also contribute. For example, various polymorphisms in FcγRIIB have been shown to lead to reduced expression on macrophages and B cells which can increase the prevalence of autoimmune conditions (103). However, the extent of TNFR mAb agonism has not been compared in these different strains. Additionally, it has been reported in individuals with the autoimmune disorder systemic lupus erythematosus that the level of FcγRIIB expression on B cells is reduced (104) further highlighting that the level of FcγRIIB expression between individuals can vary, which could impact the agonistic activity of mAb in humans.

FcγRIIB engagement however is not the only way to elicit higher-order TNFR cross-linking. In addition to FcγR cross-linking mediated agonistic activity of TNFR mAbs, it has been shown that the human IgG2 isotype can evoke greater clustering of TNFR leading to powerful receptor activation (105). Critically, this agonism is independent of the presence of FcγR and can be achieved in mice lacking all FcγR (106), although other studies indicate that hIgG2 induced agonism may be further augmented by FcγR binding (107, 108). The hIgG2 antibody is known to undergo disulfide switching in its hinge region, producing several different isoforms, including hIgG2A, hIgG2B and hIgG2A/B (109) with the hIgG2B isoform being highly agonistic and the hIgG2A isoform agonistically inert (105). Recent analysis has confirmed that the disulfide bonding pattern in the hinge region of the more agonistic isoforms gives the antibodies a less flexible conformation leading to increased agonism whereas the isoforms with higher flexibility were found to be less agonistic (110). Although initially shown first for anti-CD40 mAb, this capability of the hIgG2(B) isotype has subsequently been confirmed for OX40 and 4-1BB and also CD28 (a member of the immunoglobulin receptor superfamily) (99, 105).

Detailed characterization of several anti-TNFR mAb has also revealed that the level of agonistic activity can depend on which domain of the receptor the antibody binds to. Antibodies binding to CRD1, the membrane distal domain, of the CD40 extracellular region induced higher agonistic activity than antibodies binding to the membrane proximal domains (111). Similar to CD40, mAb binding to membrane distal domain of CD27, CRD1, were more agonistic (112). However, mAb binding to the membrane proximal CRD4 of OX40 were found to be more potent agonists than mAb binding to other CRDs (113). It should also be noted that even within a single domain, activity of antibodies may be markedly different with some far more highly agonistic dependent upon their fine epitope and also in rare cases can be independent of their isotype. For example the anti-CD40 mAb, CP870,893 binds CRD1 (111) and is highly agonistic in any isotype, whereas 341G2, which also binds CRD1, is entirely inert as a hIgG1 and hIgG4 but maximally active and super-agonistic as a hIgG2 (106). Similar observations can also be made with other TNFRs (94). Of interest, most agonistic anti-CD40 mAbs, bind in CRD1 and so do not block ligand binding. In contrast, mAbs binding within CRD2/3 block ligand binding and are less agonistic (111). This observation may support a model whereby optimal agonists bind outside the ligand binding region. However, the above mentioned 341G2 mAb blocks ligand binding but is highly agonistic, indicating this model is incorrect. This observation is supported with other TNFR family members as an agonistic TNFR2 mAb was found to completely block ligand binding but still induce strong agonism (94). These observations suggest that binding to the same epitope as the natural ligand is not a key determinant of mAb-mediated receptor agonism but rather that certain domains and epitopes might be more generally preferable for driving agonism (such as CRD1). However, as detailed above this is likely to differ for individual receptors, according to their structure and biology.

## Tolerability and response of agonistic TNFRSF targeting in clinical trials

Several agents targeting the co-stimulatory members of the TNFRSF have been tested in clinical trials. Results have demonstrated that targeting certain receptors is well tolerated whereas targeting others is limited due to toxicity. A list of the agents targeting these receptors can be found in **Table 2**, with specific examples outlined in further detail below.

**Table 2:** Co-stimulatory TNFRSF targeting agents in clinical trials.

Receptor	Drug	Modality	Clinical trial
**CD27**	Varlilumab	Human IgG1	NCT04081688; NCT03307746; NCT04941287; NCT02924038; NCT03688178; NCT03038672; NCT03617328
	MK-5890	Humanized IgG1	NCT03396445; NCT04924101; NCT04165096; NCT04165070
**4-1BB**	HLX35	EGFR – 4-1BB bispecific antibody	NCT05360381; NCT05442996
	Urelumab	Human IgG4	NCT02845323; NCT02652455
	Utomilumab	Human IgG2	NCT02554812
	YH004	Humanized IgG1	NCT05040932; NCT05564806
	ADG106	Human IgG4	NCT05236608
	ATOR-1017	Human IgG4	NCT04144842
	AGEN2373	Human IgG1	NCT04121676
	EU101	IgG1 with L234, L235 and K322 mutations	NCT04903873
	ABL503	PD-L1 – 4-1BB bispecific (Fc mutated, N299A mutation with FcγRI binding retained) human IgG1	NCT04762641
	PRS-344/S095012	PD-L1 – 4-1BB bispecific (4-1BB specific Anticalin protein), Fc silenced IgG4	NCT05159388
	GEN1046	PD-L1 – 4-1BB bispecific DuoBody (Fc silenced IgG1 antibody from human PD-L1 and humanized 4-1BB antibodies)	NCT05117242; NCT04937153
	INBRX-105	PD-L1 – 4-1BB bispecific, humanized IgG	NCT03809624
	GEN1042	CD40 – 4-1BB DuoBody, Fc silenced human IgG1 bispecific antibody	NCT05491317
	YH32367	HER-2 – 4-1BB bispecific antibody	NCT05523947
	FS222	PD-L1 – 4-1BB bispecific antibody, Fc silent human IgG1	NCT04740424
	RO7122290	FAP targeted 4-1BBL bispecific	NCT04826003
	PRS-343	HER-2 – 4-1BB bispecific (4-1BB specific Anticalin protein)	NCT05190445
	RO7227166	CD19 - 4-1BBL bispecific fusion protein	NCT04077723
	NM21-1480	PD-L1 – 4-1BB – HAS tri-specific antibody	NCT04442126
	CB307	Tri-specific Humabody targeting CD137, PSMA and HSA, not interacting with FcγR	NCT04839991
**CD40**	CDX-1140	Human IgG2	NCT05029999; NCT04491084; NCT04520711; NCT05349890; NCT05231122; NCT04616248; NCT05484011; NCT04364230
	LVGN7409	Antibody with enhanced FcγRIIB binding	NCT04635995; NCT05152212
	Mitazalimab	Human IgG1	NCT04888312
	2141-V11	Human IgG2 with enhanced FcγRIIB binding	NCT05126472; NCT04059588; NCT04547777
	SEA-CD40	Non-fucosylated humanized IgG1	NCT02376699; NCT04993677
	APX005M	Humanized IgG1	NCT03165994; NCT03389802; NCT04130854; NCT05419479; NCT03719430; NCT04337931; NCT02706353; NCT02600949; NCT03502330
	TQB2916	Humanized IgG2	NCT05213767
	RO7300490	FAP targeted CD40 bispecific agonist	NCT04857138
	SL-172154	SIRPα-Fc-CD40L fusion protein	NCT04406623; NCT05483933; NCT05275439
	MP0317	FAP - CD40 - HSA tri-specific DARPin molecule	NCT05098405
	NG-350A	Tumor selective anti-CD40 expressing adenoviral vector	NCT05165433
	LOAd703	Oncolytic adenovirus encoding trimerized CD40L and 4-1BBL	NCT03225989; NCT02705196; NCT04123470
	MEM-288	Oncolytic adenovirus encoding IFNβ and CD40L	NCT05076760
	Vaccine with tumor cells and GM.CD40L	Vaccine with cells expressing granulocyte macrophage colony stimulating factor and CD40L	NCT00101101
	HPV vaccine + anti-CD40	HPV vaccine +/- anti-CD40	NCT03418480
	CMN-001	Dendritic cell therapy, cells electroporated with RNA from tumor specimen and CD40L RNA	NCT04203901
	Selicrelumab; RO7009789*	Human IgG2	NCT03193190
**OX40**	MEDI6469	Mouse IgG1	NCT02274155
	BMS 986178	Human IgG1	NCT03831295; NCT03410901
	INCAGN01949	Human IgG1	NCT04387071
	BGB-A445	IgG1	NCT04215978
	HFB301001	Human IgG1	NCT05229601
	MEDI0562	Humanized IgG1	NCT03336606
	IBI101	Humanized IgG1	NCT03758001
	BAT6026	Afucosylated human IgG1	NCT05109650; NCT05105971
	PF-04518600	Humanized IgG2	NCT03092856; NCT03217747; NCT03971409; NCT02554812; NCT03390296; NCT03636503
	FS120	OX40 – 4-1BB bispecific, Fc silenced human IgG1	NCT04648202
	ES102	Hexavalent humanized IgG	NCT04991506; NCT04730843
	INBRX-106	Hexavalent IgG1	NCT04198766
	EMB-09	Tetravalent PD-L1 – OX40 bispecific antibody	NCT05263180
	SL-279252	PD-1-Fc-OX40L fusion protein (IgG4 Fc)	NCT03894618
	mRNA-2752	Lipid nanoparticle encapsulating OX40L, IL-23 and IL-36γ mRNAs	NCT03739931
	DNX-2440	Oncolytic adenovirus expressing OX40L	NCT04714983
**GITR**	INCAGN01876	Humanized IgG1	NCT04470024; NCT04225039
	BMS-986156	Human IgG1	NCT04021043
	REGN6569	Antibody	NCT04465487
	ASP1951	Human tetravalent antibody	NCT03799003
**TNFR2**	BI-1808	Human IgG1	NCT04752826
	SIM1811-03	Humanized IgG1	NCT05569057
	HFB200301	Antibody	NCT05238883

*was formerly CP870,893.

Modalities targeting the co-stimulatory receptors CD27, 4-1BB, CD40, OX40, GITR and TNFR2 are summarized in the table. The clinical trials which are active, recruiting or not yet recruiting are listed.

## Targeting CD27

As discussed above, CD27 is required for generating functional immune responses and targeting this receptor in pre-clinical studies has generated promising results supporting clinical evaluation. Varlilumab is a human IgG1 anti-CD27 antibody. It was well tolerated up to the maximum tested dose of 10 mg/kg with no major adverse events as a monotherapy (114, 115). Most of the toxicity related events were grade 1 or 2 with fatigue, rash, nausea, and diarrhoea the most common. Only 1 out of 56 patients had a transient grade 3 adverse event which was asymptomatic hyponatremia at 1 mg/kg. As a monotherapy, Varlilumab showed biological and clinical efficacy against tumors including hematologic malignancies, melanoma and renal cell carcinoma (114, 115). It stimulated chemokine secretion, increased the number of activated T cells and induced Treg depletion. Overall, 8 out of 56 patients had stable disease (SD) and 1 patient had a partial response (PR) (115). More recently, Varlilumab has been combined with anti-programmed cell death protein-1 (PD-1) checkpoint blockade and no additional toxicities were observed compared to anti-CD27 monotherapy. Although the initial results suggested that the combination treatment was safe and induced SD in 17% of colorectal cancers (CRC), SD in 39% of ovarian cancer (OVAC) patients, PR in 5% of CRC and PR in 10% of OVAC patients (116), more recent results revealed that the objective response rate (ORR) observed in the study was less impressive: 0% for renal cell carcinoma, 5% for CRC, 12.5% for head and neck squamous cell carcinoma and 12.5% for OVAC (117). Following promising results of a pre-clinical study demonstrating that anti-CD27 and anti-CD20 mAb in combination induced robust anti-tumor efficacy in pre-clinical B-cell lymphoma models (118), another clinical study was designed where Varlilumab was combined with the anti-CD20 antibody Rituximab to test efficacy in relapsed or refractory B-cell lymphoma. Combination treatment was in general safe but induced a grade 3 or higher adverse event in 33% of patients. The treatment was efficacious in tumors with T-cell activated status inducing SD in 3 out of 26 patients and PR in 4 out of 26 patients (119). Another CD27 targeting agonistic mAb in development is MK-5890, which is a humanized IgG1 antibody that is being tested in the clinic as a single agent or in combination with PD-1 blocking agents in advanced solid tumors. The pre-clinical characterization of the mAb demonstrated that it could induce anti-tumor responses as a monotherapy or in combination with PD-1 blockade (120). Early results suggest an acceptable safety profile, although 24% of patients in the monotherapy group developed grade 3 or 4 adverse events related to treatment. Combination treatment did not increase the level of adverse events observed with single agent. Early signs of efficacy with MK-5890 monotherapy or combination, stimulating anti-tumor responses in patients, were observed (121). Although the mAb could induce transient upregulation of chemokine levels in patients, it also induced decreases in the level of circulating T cells (120) suggesting that identifying the right dosing regimen will be important for the successful application of this mAb. A recent study in a pre-clinical setting addressed the determinants of agonism for anti-CD27 mAb (112). It demonstrated that agonism is dictated in part by the mAb-binding domain, with the membrane distal, externally facing epitopes delivering the highest level of agonism. Additionally, the agonistic activity of hIgG1 mAb was shown to be improved by Fc engineering through either enhanced binding to FcγRIIB or hIgG2 isotype selection. The anti-CD27 mAb currently in clinic (**Table 2**) are unmodified hIgG1 antibodies, likely sub-optimal for agonism, and so armed with this encouraging pre-clinical data, the next generation of anti-CD27 mAb may provide greater clinical efficacy.

## Targeting 4-1BB

4-1BB activation contributes to an optimal immune response and pre-clinical targeting of 4-1BB in mouse tumor models generated robust anti-tumor responses, supporting clinical evaluation. There are two mAbs that have been explored comprehensively in the clinic that target 4-1BB. Utomilumab is a human IgG2 antibody that has been shown to have a favorable safety profile, being well tolerated up to 10 mg/kg. The majority of the adverse events caused by the antibody were grade 1 or 2 including rash, dizziness, decreased appetite and fatigue in less than 10% of the patients in the study. Only 1 patient developed a grade 3/4 fatigue without increased transaminase levels. The overall ORR in solid tumors was 3.8% whereas the ORR in fifteen Merkel cell carcinoma patients was 13.3% with one PR and one complete response (CR) (122). Utomilumab has also been tested in combination with anti-CD20 treatment in patients with relapsed or refractory follicular lymphoma and CD20^+^ non-Hodgkin lymphoma (NHL). Initial results suggested that the combination did not affect tolerability with the majority of the treatment related adverse events being grade 1 or 2. The combination treatment showed some clinical activity especially in the NHL patients (123). Additionally, safety of Utomilumab in combination with anti-PD-1 blockade was tested in patients with advanced solid tumors and the combination was found to be tolerable with mainly grade 1 or 2 toxicities and PR or CR in 6 out of 23 patients in the study (124). However, despite tolerability, clinical responses have overall been underwhelming.

Urelumab is another 4-1BB targeting agonist antibody which is of human IgG4 isotype. A study testing the safety and tolerability of Urelumab indicated that the maximum tolerated dose (MTD) of antibody given every 3 weeks was 0.1 mg/kg and higher doses induced liver toxicity in a higher percentage of patients and at higher severity above 1 mg/kg dose (125). In another study in which Urelumab was combined with Rituximab, the MTD was again found to be 0.1 mg/kg but the combination did not enhance the effect achieved by Rituximab alone (126). Several pre-clinical studies suggested that the liver toxicity induced by agonist anti-4-1BB antibody could be due to infiltration and activation of macrophages in the liver which leads to infiltration and abnormal activation of T cells, mainly CD8^+^ T cells, leading to tissue damage (127, 128). Minimizing FcγR interactions through deglycosylation has been shown to reduce these toxicities (129).

## Targeting CD40

CD40 signaling is important for APC (DC and B cell) activation and the development of strong T-cell responses. It is one of the most targeted members of the TNFRSF in clinical trials. One of the initial antibodies to be tested in multiple studies was CP870,893 which is a human IgG2 mAb. However, the antibody had to be given at low doses due to the MTD being 0.2 mg/kg. The antibody achieved modest clinical effects as a monotherapy in advanced solid tumor patients potentially due to the low doses not saturating the receptor (16). CP870,893 has also been tested in combination with multiple agents ranging from checkpoint blockade antibodies to chemotherapy. Although a significant improvement in response was not achieved with checkpoint blockade combination, combining anti-CD40 mAb with chemotherapy achieved significant responses in pancreatic ductal adenocarcinoma patients (130). Another human IgG2 anti-CD40 mAb recently developed is CDX1140. Initial studies suggested that the antibody is tolerated up to 1.5 mg/kg as a single agent or in combination with a recombinant dendritic cell growth factor, with the majority of the adverse events being low grade and early suggestion of clinical benefit in advanced solid and hematologic tumor patients (131). The 1.5 mg/kg dose is expected to give better systemic targeting of the receptor and tissue penetration compared to the MTD of CP870,893.

As described above, human IgG2 antibodies can elicit TNFR activation without requiring FcγR mediated cross-linking. However, there is also interest in developing agents with enhanced ability to bind to FcγRIIB to mediate optimal cross-linking of the antibody, leading to greater receptor clustering and activation. APX005M is a humanized IgG1 anti-CD40 antibody possessing the S267E mutation in its Fc domain which enhances the affinity for FcγRIIB binding by 30-fold (132). Combining APX005M with anti-PD-1 blockade to treat anti-PD-1/PD-L1 refractory melanoma patients showed that the combination did not increase toxicity and the majority of adverse events were grade 1 or 2. Early results from the study are promising and indicate that the combination evokes clinical benefit (133).

## Targeting OX40

Pre-clinical studies demonstrated the anti-tumor potential of reagents targeting OX40 and agonistic anti-OX40 antibodies have been shown to be well tolerated in patients. However, the response rates as a monotherapy have been low. GSK3174998 was an agonist humanized IgG1 mAb tested against advanced solid tumors but only induced 1 PR and 1 SD in 45 patients as a monotherapy and the combination with the anti-PD-1 mAb Pembroluzimab did not significantly improve the efficacy expected with Pembrolizumab alone (134). A humanized IgG2 mAb PF-04518600 was tested as a monotherapy in advanced solid tumor patients but only 1 out of 25 patients had a PR while 15 out of 25 had SD (135). In a recent study in which PF-04518600 was combined with Utomilumab, early indications were that the combination was found to be well tolerated and 7 out of 10 melanoma patients and 7 out of 20 non-small cell lung cancer (NSCLC) patients experienced SD in addition to only 1 NSCLC patient experiencing a PR (136).

Another agonistic anti-OX40 mAb being tested in clinical trials is MEDI0562 which is a humanized IgG1 antibody. As a monotherapy in advanced solid tumors, MEDI0562 was found to be safe with the majority of adverse events being grade 1 or 2. Despite the favorable safety profile, only 2 out of 55 patients experienced a PR and 24 out of 55 patients experienced SD (137). In another study where MEDI0562 was combined with anti-PD-L1 or anti-cytotoxic T-lymphocyte associated protein 4 (CTLA-4) immune checkpoint blockade in advanced solid tumors, early results indicated that the combinations induced grade 3 or 4 adverse events in a high frequency of patients and only 11.5% of patients in the anti-PD-L1 combination group showed PRs. 34.6% of patients in the anti-PD-L1 combination group and 29% in the anti-CTLA-4 combination group experienced SD (138).

## Targeting GITR

GITR activation leads to the development of strong T-cell responses and mouse tumor model studies have demonstrated the anti-tumor potential of GITR targeting. Several agonistic antibodies targeting GITR have been tested in clinical trials. MK-1248 is an agonist humanized IgG4 antibody against GITR. In a study investigating the tolerability of MK-1248 as a single agent or in combination with anti-PD-1 blockade in advanced solid tumors, it was found that despite approximately 50% of patients in both arms of the study developing grade 3 or higher adverse events, the clinical benefit was very limited. No objective response was achieved with monotherapy and only 1 CR and 2 PRs were observed in the combination arm. 15% of patients receiving single agent experienced SD whereas 41% of patients receiving combination therapy experienced SD (139). Another agonistic anti-GITR agent is BMS-986156, which is a human IgG1 antibody. BMS-986156 was well tolerated as a single agent in advanced solid tumor patients with no grade 3 or higher adverse events and only 9.3% of patients in combination with anti-PD-1 experiencing grade 3 or 4 adverse events. Despite the favorable safety profile, no response was observed with BMS-986156 as a single agent and the highest ORR in the combination group was only 11.1% (140). MK-4166 is another human IgG1 anti-GITR antibody that has been recently tested in advanced solid tumor patients in combination with anti-PD-1 blockade. Although the treatments were found to be well tolerated, single agent again did not induce any clinical benefit. Comparing the checkpoint blockade treatment naïve versus pre-treated melanoma patients showed that the treatment naïve patients were responsive to MK-4166 and anti-PD-1 combination. 5 out of 13 patients had a CR and 3 out of 13 patients had a PR suggesting that the combination treatment might be efficacious in this particular group of patients (141).

## Targeting TNFR2

TNFR2 targeting agonist mAbs can generate strong anti-tumor T-cell immunity but are mainly still in pre-clinical development and only recently starting clinical assessment. MM-401 is an agonist anti-human TNFR2 mAb in development. Using a mouse surrogate version of the antibody, it was found that TNFR2 agonism could generate strong anti-tumor responses by activating CD8^+^ T cells and NK cells with activity dependent on FcγR interactions, presumably mediated by cross-linking of the receptor. In addition, the antibody synergized with checkpoint blockade (142). BI-1910 is another agonist anti-TNFR2 mAb in development following promising results from a surrogate anti-mouse TNFR2 antibody; this mAb induced strong anti-tumor responses in several pre-clinical tumor models and was effectively combined with checkpoint blockade antibodies. The dominant mechanism of action was expansion of CD8^+^ T cells and improved CD8^+^ to Treg ratio in the tumor site (143). BI-1808 is an alternative TNFR2 targeting mAb, classified as a deleting, ligand blocking molecule. However, pre-clinical studies with a mouse surrogate indicated intra-tumoral Treg depletion and effector T-cell expansion leading to an improved CD8:Treg ratio. Similar results were obtained with BI-1808 in pre-clinical characterization. BI-1808 was found to be well tolerated in non-human primates and is in clinical assessment (143, 144). HFB200301 is also an anti-TNFR2 agonist antibody which is already in a phase I clinical trial of advanced solid tumor patients (145). Using human TNFR2 knock-in mouse models, it was suggested that HFB200301 could stimulate anti-tumor responses through expansion of effector T cells and NK cells without depleting the Tregs. The agonistic ability of the antibody was found to be independent of FcγR mediated cross-linking (146). Although much of the data is not yet mature, with peer review lacking for most of the pre-clinical studies, the potential of TNFR2 targeting antibodies in oncology are exciting and the initial results from clinical trials are eagerly awaited by the immuno-oncology community.

## Recent approaches in targeting TNFRSF members to overcome current limitations

### Fc engineering

As described above, despite success in pre-clinical studies, clinical efficacy of targeting TNFRSF members has been limited. One factor which may help to explain this is the lack of a human antibody isotype equivalent of mIgG1 with preferential binding toward FcγRIIB to facilitate agonistic activity. Therefore, in order to enhance FcγRIIB engagement, Fc engineering approaches have been developed to improve the affinity of antibodies toward hFcγRIIB. Although several mutations such as SE (S267E) and SELF (S267E-L382F) have been identified to improve affinity to hFcγRIIB, those mutations improved affinity to hFcγRIIa as well, due to sequence and structural similarity between the two receptors. Other mutations such as V9 (G237D-P238D-P271G-A330R) and V11 (G237D-P238D-H268D-P271G-A330R) however, were found to specifically improve the affinity of antibodies toward hFcγRIIB by approximately 32 and 96 fold, respectively (107). Comparing WT and Fc engineered anti-human CD40 antibodies in mice expressing hFcγRs, the variant with the V11 mutation was found to be superior to others, indicating the possibility of this approach to be taken forward for further development. Subsequent analysis demonstrated that systemic delivery of the agonistically enhanced variant could pose a risk of inducing toxicity and optimal receptor occupancy might not be reached with the MTD. Delivering the mAb *via* intra-tumoral injections was shown to ameliorate toxicity, yet retain significant tumor control even at low doses (147) indicating that where this method of delivery is practical (e.g. for localized/accessible lesions) it could provide a solution.

Another approach to overcome the requirement for mAb cross-linking could be *via* alternative, FcγR-independent, Fc domain engineering which was recently demonstrated for anti-human OX40 mAbs. Building on seminal studies showing that E345R, E345K and E430K single point mutations in the Fc region could promote “on-target” multimerization (once the mAb binds to the receptor) of the mAbs to facilitate optimal engagement of the hexa-headed C1q molecule (148, 149), Zhang et al. showed that E345R single mutation or K248E-T437R double mutations in the Fc region could induce “on-target” multimerization of agonistic OX40 antibodies, leading to activation of the receptor in an FcγR-independent way (150, 151). Although the Fc engineered antibodies were active in the absence of FcγR cross-linking, their activity could be further improved by FcγRIIB mediated cross-linking, suggesting that this approach could provide the possibility of targeting receptors in tissues without FcγRIIB availability but when FcγR are available, the activity will be further boosted.

### Receptor cross-linking independent of FcγR

Although improving FcγRIIB affinity of antibodies can augment agonism, as previously mentioned the availability of FcγRIIB at the relevant anatomical site to provide the cross-linking can be a limiting factor. Thus, alternative approaches have been developed to generate agonistic agents without the requirement of FcγR mediated cross-linking. In addition to the hIgG2 isotype, soluble recombinant TNFSF ligands have been explored as a means to replicate the natural multimeric ligand-receptor interaction. The potency of soluble trimeric ligands could be improved by additional cross-linking and this approach was demonstrated for several ligands including OX40L, CD40L and 4-1BBL (13, 152). However, as the soluble trimeric ligands still require additional cross-linking, practicality of this approach *in vivo* is likely to be challenging due to possible short serum persistence of the trimers and also additional non-native sequences potentially making the products more immunogenic. To overcome this limitation, multimeric forms of soluble trimeric TNFSF ligands such as Fc fusion proteins have been developed. Multimeric ligands do not require the additional cross-linking required by the trimeric forms and the Fc fusion facilitates better *in vivo* persistence *via* its interaction with the neonatal Fc receptor (FcRn) (153). A CD27L-Fc fusion protein designed to mimic the natural CD27L activity was found to be active in *in vitro* and *in vivo* assays boosting T-cell activation (154). In that study, one CD27L extracellular domain (ECD) was fused to one Fc domain suggesting that the active product consisted of multimeric trimers of the ligand and multimers of Fc domains. More recently, a hexameric human CD27L fusion protein consisting of six CD27L ECDs and a silent human IgG1 Fc domain (not interacting with FcγR) has been reported (155). In this construct, three ECDs of the ligand are linked in a single chain format and fused to the IgG1 Fc domain with the idea of bringing two ligand trimers together upon Fc domain dimerization (**Figure 2B**). The fusion protein induced activation and proliferation of T cells in *in vitro* and *in vivo* experiments independently of FcγR engagement (155). Additionally, the hexameric fusion protein demonstrated anti-tumor efficacy in pre-clinical models. Hexameric Fc fusion ligand proteins in the same format have also been developed for CD40L, GITRL and 4-1BBL (156–158). Despite the promising pre-clinical results, the hexameric ligand proteins have short half-lives in circulation. Although this could be considered as a disadvantage, shorter stimulation of the immune cells can also lead to generation of a strong response and possibly could be better than chronic stimulation, which might have detrimental effects (159, 160). It has been shown in multiple studies that continuous stimulation of CD27 leads to defects in the immune cells. Continuous stimulation of CD27 by constitutive expression of CD70 on B cells resulted in increased apoptosis and depletion in NK cells (161) or T-cell immunodeficiency (159). Similarly, continuous 4-1BB stimulation leads to overactivation of CD8^+^ T cells and macrophages which eventually results in impaired CD8^+^ T-cell activity (160). Thus, timing and strength of stimulation are crucial in inducing a strong immune response and avoiding immunopathology. By experimentally determining the correct dose, schedule and treatment routes the hexameric ligands might generate strong immune responses in patients. On the other hand, it is worth noting that agonistic ligand formats, specifically TNFR2 specific recombinant TNF ligand protein, have also been developed with the aim of expanding Tregs in non-cancer contexts. A nonameric version of the recombinant protein was initially found to have suboptimal serum retention *in vivo* but a newly developed version in which an Fc silent irrelevant IgG molecule is fused to two trimeric ligand units to generate a hexameric ligand showed improved pharmacokinetics and robust Treg expansion *in vivo* (162).

Recent technological advances in the field have enabled the use of computational methods to design desired structures. Using such approaches, researchers have produced antibody molecules in various oligomeric states, in a format described as “antibody nanocages”. These nanocages were found to activate several receptor targets, including converting an antagonist anti-CD40 mAb into an agonist due to the ability of the designed structure to induce receptor clustering (163). This approach could potentially be applied to a plethora of different receptors to identify the best design for optimal receptor activation in each case.

### Reagents targeting tumor microenvironment to induce localized TNFR activation and reduce toxicity

In addition to the variation of the availability of FcγRIIB in target tissue to provide optimal cross-linking of agonistic mAbs, off-target toxicity has also been an issue. Although some agonistic mAbs such as Varlilumab against CD27 was well tolerated, the clinical efficacy was modest. In contrast, the 4-1BB agonist Urelumab was active but found to induce liver toxicity at high doses. The mechanism behind the toxicity of Urelumab is thought to be the activation of the liver resident FcγR-expressing Kupffer cells, with the agonistic cross-linking of the anti-4-1BB mAb enabled by the high level of FcγR expressed on these myeloid cells (98) or other FcγR-expressing cells in the liver, such as FcγRIIB expressing sinusoidal liver endothelial cells (164). Activated Kupffer cells produce IL-27 which is an inflammatory cytokine involved in infiltration and expansion of other immune cells, especially T cells into the tissue (127). Hepatotoxicity following 4-1BB agonism indicated that systemic delivery of the agonistic reagents has the risk of off-target toxicity. Thus, recent efforts have focussed on eliminating the risk associated with systemic delivery in favor of targeted agonism – localizing the mAb to the desired site. One approach has been to generate recombinant proteins with a tumor targeting domain. For example, single chain fragment variable (scFv) domains of an anti-4-1BB mAb have been fused to a trimerization domain (producing a trivalent 4-1BB targeting molecule) with further fusion of a tumor targeting domain on the C-terminus to direct the trimer to the tumor site (165). Although the trimeric protein had short *in vivo* stability, the anti-tumor response generated in mouse tumor models was similar to an agonistic anti-4-1BB mAb and the trimer did not induce toxicity, which was apparent with the agonistic mAb. Additionally, repeated dosing of the trimeric protein also did not induce off-target toxicity indicating that targeted agonism approach could overcome the non-specific toxicity.

More recently, a tumor antigen targeting 4-1BB bispecific molecule was generated with one arm of the antibody designed to target a tumor antigen and the other designed to form a trimeric h4-1BBL. The bispecific molecule was generated in an Fc silent format to maintain normal antibody-like pharmacokinetics but at the same time eliminating FcγR engagement to prevent off-target toxicity. Binding of the tumor antigen specific arm at the tumor site allows accumulation of 4-1BBL in the tumor tissue to facilitate multimeric interaction between the ligand and receptor (**Figure 2C**) and activate the T cells in the tumor microenvironment. The bispecific molecule had a favorable pharmacokinetic profile and could accumulate in the tumor site, confirmed in non-human primates (166). Additionally, the bispecific molecule proved to be able to induce activation of T cells from human tumor tissues and also induce anti-tumor immunity in pre-clinical models. However, the main activity was observed when the bispecific molecule was used in combination with another T-cell bispecific agent stimulating the TCR and targeting a tumor antigen (166, 167), indicating that optimal co-stimulation happens in the presence of TCR stimulation. While the bispecific molecule had favorable serum stability, it did not induce toxicity indicating that it could be used in combination with other T-cell inducing treatments. Similar bispecific molecules with a scFv arm targeting a tumor associated antigen and a TNFSF ligand arm targeting a co-stimulatory receptor on the T cells have also been characterized in other studies (168). In these molecules however, a tag was inserted for purification purposes and its immunogenicity will have to be assessed further during *in vivo* validation of these reagents.

Another approach to induce TNFR clustering involves duokines, where both arms of the bispecific molecule are targeting members of the TNFRSF. Initially, the proteins were developed by either fusing one ECD protomer of a TNFSF ligand to one ECD protomer of another TNFSF ligand to allow trimerization of the ligand molecules by non-covalent interactions or by developing them as a single chain polypeptide in which three ECDs of each ligand were linked onto the same polypeptide chain separated by flexible linkers (169). Depending on the choice of ligands, this approach allows targeting of receptors in cis (on the same cell surface) or trans (on different cells) orientations. The single chain duokines were found to be more stable than non-covalently formed duokines and could induce *in vitro* and *in vivo* stimulation of T cells as co-stimulatory molecules. Using 4-1BBL-CD40L as a trans acting duokine or 4-1BBL-CD27L as a cis acting duokine, Fellermeier-Kopf and colleagues showed that both molecules could induce anti-tumor immunity in a pre-clinical melanoma model in combination with a TCR targeting bispecific antibody (169). In a subsequent study, Fc fusion proteins of the duokines were generated to facilitate enhanced stability in circulation with the active protein adopting an antibody structure with each single chain trimeric ligand domain being fused to Fc regions and dimerization of the Fc regions bringing two trimeric ligands together (170). Although the protein was still active in combination with a TCR targeting bispecific antibody, interestingly, the Fc fusion did not improve the pharmacokinetic profile. These data demonstrated the possibility of using these duokines to target two co-stimulatory TNFR molecules to boost the anti-tumor response. By identifying the optimal combination strategies, they could potentially enhance the anti-tumor responses in the clinic.

It has been clearly observed that blocking the immune checkpoint molecules PD-1 or CTLA-4 can generate strong anti-tumor responses but the majority of the patients are either refractory or develop resistance to these therapies. In recent studies, bispecific molecules targeting the checkpoint inhibitory receptors and co-stimulatory members of the TNFRSF have been developed as a means to enhance their activity. There are multiple advantages to this approach: First, the interaction of the inhibitory checkpoint receptor and its ligand is blocked to release the suppression on the immune response. Second, the inhibitory molecules are mainly expressed in the tumor microenvironment and this ensures targeted activation of the co-stimulatory receptor at the tumor microenvironment, avoiding systemic toxicity. Third, the bispecific antibody can be generated in an Fc silent format to avoid potential systemic toxicity with co-stimulatory receptor clustering achieved by the checkpoint receptor targeting arm acting as an anchoring domain. An Fc silent IgG1 bispecific antibody in a tetravalent format with two Fab arms targeting PD-L1 and two 4-1BB targeting domains introduced into the CH3 domains, termed Fc-region with antigen binding, was recently developed (**Figure 2D**). The mouse surrogate version of the bispecific induced activation of T cells *in vitro* and induced anti-tumor immunity *in vivo* without hepatotoxicity. The human version of the protein induced human T-cell activation *in vitro* and toxicology studies in non-human primates, enabled by cross-reactivity between species, showed that the bispecific was well tolerated (171) and had higher activity than the combination of the single agents. Similar bispecific molecules in tetravalent formats targeting PD-L1 and CD40 or 4-1BBL have also been reported in other studies. *In vitro* characterization of these products showed PD-1/PD-L1 blockade and target receptor activation in an FcγR independent manner, supporting further validation in *in vivo* studies (172). In support of these findings with bispecific molecules, it was recently shown that an anti-PD-1/GITRL bispecific molecule induced a different mechanism of action than the combination of single agents and was more efficacious in pre-clinical studies (9). The co-stimulatory antibodies being tested in combination with checkpoint blockade antibodies to date have shown favorable tolerability in clinical trials (see above), and the recent findings support the idea that the bispecific molecules could achieve better results than the combination treatments.

## Conclusion

TNFRSF members represent powerful targets for immunomodulation. Promising pre-clinical data of agonistic mAbs targeting TNFRSF has clearly demonstrated their potential to provide anti-tumor efficacy. However, the translation from the pre-clinical studies to the clinic has been difficult and lack of significant response rates or toxicity in the clinic with conventional mAbs has directed researchers to develop new strategies.

Other immunomodulatory agents such as the checkpoint blockade antibodies have shown better success than the agonistic antibodies against TNFRs. However, while the responses thus far are limited, there is an opportunity for combining the two strategies, as has been shown in pre-clinical studies. With new approaches, such as targeted agonism and bispecifics delivering two or more different mechanisms of action with a single agent, success rates may improve. The challenge however remains the same – evoking powerful, curative immune responses while avoiding toxicity. Hopefully, such innovation will finally unlock TNFR targeting for the clinic.

## Author contributions

OD researched data and wrote the manuscript with MC, OD, and AE produced the figures. All authors contributed to the article and approved the submitted version.
